# Supplementary material for: Predicting Species Boundaries and Assessing Undescribed Diversity in Pneumocystis, an Obligate Lung Symbiont
Source: J Fungi (Basel). 2022 Jul 29;8(8):799. doi: 10.3390/jof8080799 (PMC9409666; doi:10.3390/jof8080799)
Supplement: Supplementary file 1 [file jof-08-00799-s001.zip › Supplementary_Table_2.pdf]

Supplementary Table 2 Support for delimitation hypotheses from BPP analysis

Delimitation Hypothesis	*Rodents* Posterior Probability	*Bats* Posterior Probability	*Primates* Posterior Probability
ABGD	0.000	0.000[b]	0.000
GMYC	0.000	0.000	0.000
PTP	0.000	0.009	0.899
BPP	0.993	0.902	0.899

[b]ABGD inferred one species that was paraphyletic with respect to the MCCT (*Felis catus* 1 + *Sus scrofa domesticus 1*) that could not be evaluated by BPP. The ABGD delimitation without this paraphyletic collapse received no support.
